# Three-Dimensional Geometric Morphometric Characterization of Facial Sexual Dimorphism in Juveniles

**DOI:** 10.3390/diagnostics15030395

**Published:** 2025-02-06

**Authors:** Riccardo Solazzo, Annalisa Cappella, Daniele Gibelli, Claudia Dolci, Gianluca Tartaglia, Chiarella Sforza

**Affiliations:** 1 Laboratory of Functional Anatomy of the Stomatognathic System (LAFAS), Department of Biomedical Sciences for Health, University of Milan, 20133 Milan, Italy; 2 U.O. Laboratory of Applied Morphology, IRCCS Policlinico San Donato, 20097 San Donato Milanese, Italy; 3 Department of Biomedical Sciences for Health, University of Milan, 20133 Milan, Italy; 4 Department of Biomedical, Surgical and Dental Sciences, University of Milan, 20122 Milan, Italy; 5 Fondazione IRCCS Cà Granda, Ospedale Maggiore Policlinico, 20122 Milan, Italy

**Keywords:** sexual dimorphism, geometric morphometrics, three-dimensional imaging, maxillo-facial district, growth trajectory, spatially dense geometric morphometrics

## Abstract

**Background:** The characterization of facial sexual dimorphic patterns in healthy populations serves as valuable normative data to tailor functionally effective surgical treatments and predict their aesthetic outcomes and to identify dysmorphic facial traits related to hormonal disorders and genetic syndromes. Although the analysis of facial sexual differences in juveniles of different ages has already been investigated, few studies have approached this topic with three-dimensional (3D) geometric morphometric (GMM) analysis, whose interpretation may add important clinical insight to the current understanding. This study aims to investigate the location and extent of facial sexual variations in juveniles through a spatially dense GMM analysis. **Methods:** We investigated 3D stereophotogrammetric facial scans of 304 healthy Italians aged 3 to 18 years old (149 males, 155 females) and categorized into four different age groups: early childhood (3–6 years), late childhood (7–12 years), puberty (13–15 years), and adolescence (16–18 years). Geometric morphometric analyses of facial shape (allometry, general Procrustes analysis, Principal Component Analysis, Procrustes distance, and Partial Least Square Regression) were conducted to detail sexually dimorphic traits in each age group. **Results:** The findings confirmed that males have larger faces than females of the same age, and significant differences in facial shape between the two sexes exist in all age groups. Juveniles start to express sexual dimorphism from 3 years, even though biological sex becomes a predictor of facial soft tissue morphology from the 7th year of life, with males displaying more protrusive medial facial features and females showing more outwardly placed cheeks and eyes. **Conclusions:** We provided a detailed characterization of facial change trajectories in the two sexes along four age classes, and the provided data can be valuable for several clinical disciplines dealing with the craniofacial region. Our results may serve as comparative data in the early diagnosis of craniofacial abnormalities and alterations, as a reference in the planning of personalized surgical and orthodontic treatments and their outcomes evaluation, as well as in several forensic applications such as the prediction of the face of missing juveniles.

## 1. Introduction

Sexual dimorphism refers to the phenotypic differences in morphology, physiology, and behavior characterizing males and females of the same species [1,2]. A trait is defined as ‘sexually dimorphic’ when it exhibits average differences between the two sexes [3]. Overall, sexual dimorphism is the result of a complex developmental process where males and females differentiate over time. Humans are characterized by more subtle manifestations of sexual dimorphism compared to other primates [3,4], and the most extensively studied dimorphic trait is the craniofacial district [5] as it exhibits a certain degree of sexual dimorphism [6], although to a lower extent compared to other traits such as the pelvis [3,7,8]. The faces of males and females differ in overall size and shape and in the proportions between constituting regions like the forehead, eyes, nose, cheeks, lips, and chin [4,9,10,11,12,13]. Overall, compared to females, males are characterized by relatively shorter midfaces, longer and larger lower facial thirds [14], more oblique foreheads, more prominent supraorbital ridges and glabella, larger noses and nasal humps, greater projection of the oral and mental regions, and more angular and wide jaws [4,15,16,17,18,19]. These differences are particularly evident in adults. However, as sexual dimorphism changes as a function of age [20,21], the visible facial sexual dimorphic characteristics described in adults may not be generalizable to the face of subadults.

The sexual dimorphism of the craniofacial district becomes apparent between birth and adulthood, and the most evident changes occur with the onset of puberty [5,21]. Nevertheless, several studies have reported the appearance of sexual dimorphism prior to puberty [4,16,22,23], and one even as early as 1 year old [24]. Indeed, human sexual dimorphism is multifactorial in nature: the genome (e.g., genetics, epigenetics, and genomic regulators) and the environment (e.g., endogenous (hormones) and exogenous factors (environmental exposure, socio-economic status, cultural practices, and background) are the two main interacting factors [25]. The hormone levels may influence the facial shape differences between males and females [26,27,28,29]. In utero hormones model the facial architecture, which, in turn, is developed during puberty [24] when increased testosterone levels in males induce masculinization, whilst higher levels of estrogen induce feminization of the facial shape [26,27,28,29]. The effect of in utero hormone levels on the facial architecture and sexual dimorphism of this anatomical structure could be analyzed if sufficiently sensitive methods were employed [24].

Sexual dimorphism of the craniofacial district and its development, particularly in juveniles, are of interest for evolutionary biology to detect genetic and evolutionary clues driving the morphological differences in the two sexes, the biological factors contributing to them, their inheritance, and their evolutionary advantages [2,5,30]; to cognitive sciences and social and psychological research to understand how adults and peers perception impact socialization, gender identity development, and self-perception [31,32,33,34]; to anatomy and physical and forensic anthropology [35,36,37,38] as pediatric facial dimorphism data hold forensic value, contributing to facial recognition, age estimation, and identification in forensic anthropology [21,39]; and to plastic and reconstructive surgery [40,41], dentistry [42,43], and clinical genetics and dysmorphology [44,45,46], where age- and sex-specific craniofacial norms guide diagnosis and treatment. In the latter fields, such insights are crucial for assessing facial development, identifying potential dysmorphologies, and understanding the onset and progression of sex-specific traits in children.

Sexual differences in the hard and soft tissues of the craniofacial district have been deeply investigated using morphometric assessments of anatomical structures: scoring systems were classically adopted by the anthropological field [38,47,48], while metrical assessments are traditionally used by in vivo anthropometry and in two-dimensional (i.e., photographs, X-rays, cephalograms) or three-dimensional settings [49,50,51,52]. Thus, the quantification of the craniofacial complex size and shape has been initially performed by measuring linear and angular distances between discrete and biologically meaningful landmarks [24]. However, this approach had several drawbacks as it does not capture the spatial arrangement and geometrical relationship of the structures, which are instead described by few landmarks, nor the complex morphologies, which have unevenness and curves [53].

Today, 3D imaging technologies are replacing direct anthropometry as the elective method to obtain quantitative information about facial soft tissues [54,55,56], and because of their advantage, they allow for the collection of normative 3D data in healthy control subjects, which are important for comparative studies of growth pattern, and to evaluate treatment outcomes in infants with congenital malformations and anomalies [54,57]. Weinberg et al. [57] argued that “3D imaging methods required 3D normative data”. The widespread use of these technologies in clinical practice, routinely performed as they are considered the gold standard by some [43], has advanced the analysis of craniofacial sexual dimorphism because it allowed researchers to investigate anthropometric parameters such as 3D surfaces and volumes more accurately, thus helping to further characterize the morphological and dimensional differences existing between males and females [11,51,58,59,60]. In addition, these techniques represent complete structures and surfaces, which allow researchers to develop more comprehensive analysis methods as geometric morphometric (GMM) approaches [61]. Geometric morphometric analysis may be based either on sparse or quasi-landmarks [62]. Sparse landmarks are discrete anatomical points on the craniofacial structures [63], while quasi-landmarks are densely sampled points representing the entire surface [62]. Sparse landmark methods are well-established and widely used, even though they provide limited information about landmark-deficient regions, e.g., cheeks and forehead [62]. Quasilandmark methods offer a more comprehensive analysis by capturing detailed surface information and subtle variations in the entire craniofacial district.

Although several studies have reported data on sexual dimorphism of subadults by means of anthropometry or 3D-based measurements [16,64,65,66], only recently have GMM approaches been applied to address this issue [4,10,11,22]. Kesterke et al. [4] argued that most of the studies dealing with the assessment of pediatric craniofacial sexual dimorphism fail because the age ranges under analysis are limited, few measurements are performed, and the sample sizes are small. To partially fill this gap, the present study aims to systematically investigate facial sexual dimorphism in Italian subadults using a spatially dense GMM approach. Our objectives are to identify the regions where sexually dimorphic traits are most prominent, determine when these differences emerge, and quantify the extent to which biological sex contributes to facial morphology across different developmental stages. By analyzing the entire facial surface in pediatric subjects, this study seeks to provide a more accurate, age-appropriate reference to understand pediatric facial development and sex differences, addressing a significant gap in the existing literature.

## 2. Materials and Methods

The study sample was retrospectively selected from the database held at the Laboratorio di Anatomia Funzionale dell’Apparato Stomatognatico (LAFAS) of the University of Milan, which includes 3D facial scans of more than 2000 subjects. The participants were scanned using the Vectra M3 (Canfield Scientific, Parsippany, NJ, USA), a fast (<1.5 ms), safe, and non-invasive optical stereophotogrammetric instrument [67,68,69]. The scanning procedure was conducted according to the laboratory protocol [67,68], with the subjects seated facing the instrument with a neutral expression, looking straight ahead with the eyes opened and the mouth gently closed.

To achieve the purposes of this study, only the 3D images of subjects younger than 18 years old were examined after a quality check that excluded images of poor quality (e.g., mesh interruptions, stitching errors, irregular surfaces) or in cases of non-neutral expressions. Additional exclusion criteria were geographical origins other than Italian, pre-existing traumas and pathologies involving the craniofacial district, and the presence of facial hair.

The participants’ 3D scans were categorized into four age groups representing four different craniofacial growing phases according to Slavkin [70]: early childhood (3–6 years), late childhood (7–12 years), puberty (13–15 years), and adolescence (16–18 years).

This retrospective study is part of a broader project approved by the local Ethics Committee of the University of Milan (protocol 19/24), and it was conducted according to the Declaration of Helsinki [71]. The informed consent form was signed by all volunteers or by their parents/legal guardians for underage participants.

### 2.1. Facial Mapping

**Figure 1:**
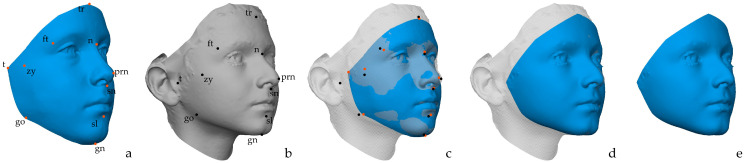
(**a**) Template and (**b**) target mesh with the annotated landmarks represented by the orange and black dots, respectively (tr: trichion; n: nasion; prn: pronasale; sn: subnasale; sl: sublabiale; gn: gnathion; ft: frontotemporale; zy: zygion; t:tragion; go: gonion); (**c**) rough alignment based on landmarks and rigid registration to approach the two meshes and to match the translation, rotation, and scaling of the template with those of the target; (**d**) non-rigid registration where the template is modified to represent the target; (**e**) final representation of the target after fine alignment of the template.

**Figure 2:**
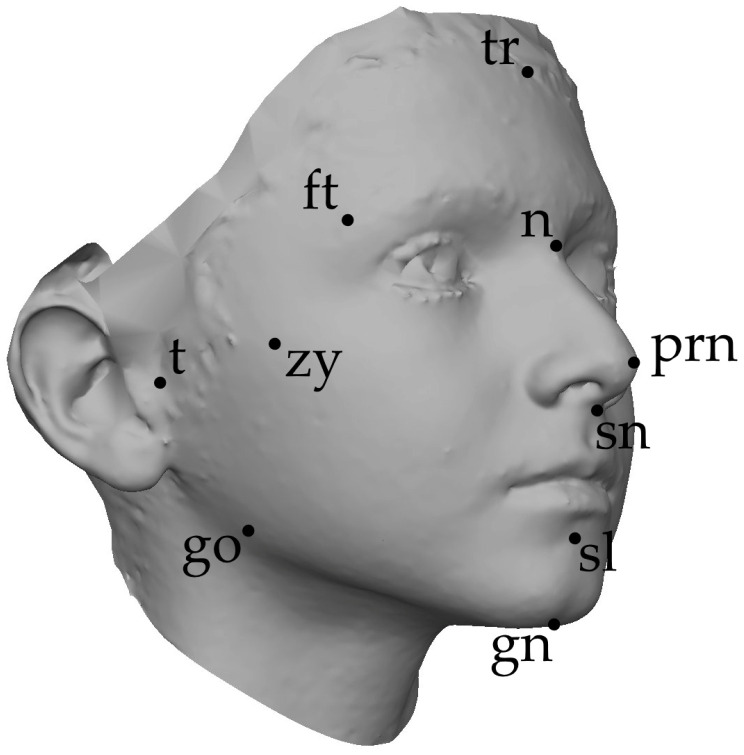
Position of the digitized anatomical landmarks (tr: trichion; n: nasion; prn: pronasale; sn: subnasale; sl: sublabiale; gn: gnathion; ft: frontotemporale; zy: zygion; t:tragion; go: gonion).

The selected 3D surface images were placed into dense correspondence to make them represented by a homologous set of points, defined as quasi-landmarks, that anatomically correspond across all images. “Homologous” means that each quasi-landmark occupies the same position relative to all the others [44], and they are defined as “quasi” since they may or may not have a well-defined biological meaning, but altogether they provide an exhaustive representation of the entire surface of interest [24]. The dense correspondence between 3D images allows the combination of multiple 3D images to obtain meaningful insights [45]. The dense correspondence between images was achieved using the MeshMonk image processing pipeline [72], which non-rigidly aligns a template, also called anthropometric mask, onto the 3D facial surfaces of the participants, also known as target surfaces. A comprehensive explanation of the pipeline to perform the non-rigid alignment and obtain dense correspondence between 3D images can be found in the original article proposing the method [72], but we briefly report the key steps visually depicted in Figure 1: (1) an initial rough alignment of the template with the target 3D models achieved through the same landmarks indicated on both surfaces (Figure 1a–c); (2) a scaled rigid alignment between the template and the target 3D models based on the ICP algorithm to remove differences in rotation, translation, and scaling between template and target (Figure 1c); and (3) a non-rigid alignment that alters and deforms the shape of the template to match that of the target surface (Figure 1d,e). Steps 1 and 2 are the preparatory ones, while the third one is the actual non-rigid alignment that Claes et al. [73] compared to “physically fitting an elastic mask onto a solid facial statue through the alignment and deformation of corresponding features onto each other”. Although the MeshMonk toolbox provides a facial template, a new one was specifically created for this study to include some critical facial regions otherwise not included, such as the gonia, the superior portion of the forehead, and the most posterolateral portion of the face. To create the new template, the 3D facial scan of a random subject was chosen as starting point. Preprocessing in the Vectra Analysis Module Software (VAM, version 7.4.6, Canfield Scientific, Parsippany, NJ, USA) was performed to remove confounding factors such as the hairs, ears, and neck to obtain only the face with all the areas of interest. The trimmed face was then imported into Blender (version 4.1, Blender Foundation, Amsterdam, The Netherlands) [74] and symmetrized over the *x*-axis to obtain a perfectly bilaterally symmetrical mesh. The mesh was decimated to reduce the vertex count without losing shape information to 7400 vertices and then iteratively modified to obtain a perfectly bilateral template mesh with vertices with a fixed edge length. The newly obtained mesh was then aligned to eight subjects randomly selected (one per each sex and age category), and the average mesh was calculated to generate the final “synthetic” template not representative of a real subject of a specific age or sex.

As the first step of the non-rigid alignment requires the indication of landmarks on the template and the target surfaces, all the selected 3D facial images included in this study were manually annotated with fourteen anthropometric landmarks, as defined by Farkas [49], and were listed and defined in Table 1 and portrayed in Figure 2.

The selected 3D facial images were exported and saved in wavefront.obj file format, and the related landmark coordinates are .txt files. These were used to accomplish the non-rigid registration implemented in MATLAB 2023b (version 23.2.0, The MathWorks Inc., Natick, MA, USA) [75] by using the code provided by the MeshMonk toolbox. The non-rigid alignment of the template on the target 3D scans included in this study required an average time of 2 min per mesh. After alignment with the template, a visual check was performed, and the failed registrations were excluded. Subsequently, the obtained quasi-landmark configurations were symmetrized by reflecting a copy of the configuration along the *x*-axis, superimposing the two configurations (original and mirrored) through Procrustes superimposition and averaging corresponding landmarks [9,76]. The symmetrization of the 3D dense configurations was necessary since the face has the intrinsic characteristic of being bilaterally symmetric and sex differences could exist both in facial symmetry and asymmetry [9,17], and for this reason, the analysis should be performed to separate the two components [9] into the symmetric and asymmetric ones. Although some important clues about sexual dimorphism can be derived from the asymmetric component [9] by applying protocols such as the one proposed by Claes et al. [9] or Zhu et al. [77], in this study, only the symmetric component has been analyzed as already carried out in previous studies [24,78,79]. This choice was driven by the biological relevance of the symmetric components, which reflect genetic and developmental factors underlying sexual dimorphism, while asymmetric variations arise from environmental and developmental noise. Furthermore, in juveniles, transient asymmetries related to the ongoing development may exist, potentially obscuring the patterns of sexual dimorphism, and this makes the symmetric component a more reliable indicator.

### 2.2. Spatially Dense Geometric Morphometrics (GMM) and Statistical Analysis

The intra-operator and inter-operator reliability in the identification of the fourteen anthropometric landmarks was evaluated in a subset of 20 participants randomly selected from the original sample. The first operator identified the landmarks twice, at least two weeks apart, while the second operator performed it once for each 3D image. The position and orientation of the 3D images were not modified, so the reliability of positioning each landmark was determined on the raw coordinates through the intraclass correlation coefficient (ICC) for the three axes separately (x, y, and z), as performed in previous studies [80,81,82]. The ICC results were interpreted according to Koo and Li [83]. Additionally, the error associated with multiple alignments of the template onto the same images was evaluated in the same subset. Specifically, each of the 20 3D images was non-rigidly aligned using the three different landmark configurations as starting points: two of the first operator and one of the second operator. A Root Mean Square Error (RMSE) was calculated between the quasi-landmarks’ configurations obtained starting from the indications of the same or of two different operators.

The symmetric quasi-landmark configurations of all were aligned to the sample mean through Generalized Procrustes Analysis (GPA) to eliminate differences in position orientation, and they were also scaled to the unit centroid size (CS) to remove size as variable [61]. The centroid size is defined as the square root of the sum of squared distances of all landmarks from their centroid, whose location is obtained by averaging the coordinates of all quasi-landmarks [84], and the centroid size of each participant was recorded and used as the size variable. To evaluate the impact of sex on facial size, the normal distribution and homogeneity of variance of the males’ and females’ centroid sizes were verified and, according to the results, a Student’s *t*-test or Mann–Whitney U test (α = 0.05) was used to assess statistically significant differences in the size of males and females faces.

To describe the major modes of shape variation and reduce data dimensionality, a Principal Component Analysis (PCA) on the Procrustes coordinates of facial shape was performed, coupled with parallel analysis to identify the number of significant principal components (PC) contributing to facial morphology. To evaluate the statistical significance of each PC, a parallel analysis was conducted using 10,000 permutations. The significant PCs were regressed onto the centroid size to evaluate the static allometry and, thus, the influence of size on the shape [85].

Sexual dimorphism of facial shape was initially assessed by computing the Procrustes distance between the average male and female 3D surfaces. The Procrustes distance is defined by Mitteroecker et al. [86] as a measure of the shape difference between two landmark configurations. The significance of the Procrustes distance was evaluated through a 10,000-permutations-based test. Lastly, a Partial Least Square Regression (PLSR) analysis was used to evaluate the effect of sex (centroid) size and their interaction with facial shape. PLSR defines the vector through the point cloud that best captures the covariance between predictors (e.g., sex or size) and response variables (the Procrustes coordinates of shape) [24,78,87]. In the present study, sex, centroid size, and their interaction were included as predictors in the PLSR model to evaluate their independent effects on shape. The effect size of each predictor was estimated through partial R^2^ [37], which explains how much of the variance is explained by the predictor, and its significance was assessed through permutation-based testing. All statistical analyses were performed in MATLAB 2023b (version 23.2.0, The MathWorks Inc., Natick, MA, USA) [75], which also handled the images.

## 3. Results

The study sample consisted of 3D facial images of 304 (149 males and 155 females) healthy Italian subadults aged 3 to 18 years old. Table 2 reports the distribution of males and females in the defined age classes, their mean age, and the pertinent *p*-values, which always found no significant differences in the mean age of participants in all age classes.

The intraclass correlation coefficient (ICC) proved that the landmarks used to initialize the non-rigid alignment can be reliably identified by the same operator twice or by two different operators (Table 3). Indeed, all ICC values are greater than 0.900, which defines “excellent” reliability according to Koo and Li [83].

To evaluate the potential effects and errors on the final aligned meshes due to the manual placement of landmarks that initiate the non-rigid alignment, we calculated the RMSE between the resulting aligned meshes (quasi-landmark configurations). The average RMSE for the quasi-landmark configurations obtained starting from the landmarks of the same operator was 0.13 mm, while that obtained when starting from the landmarks of different operators was 0.16 mm.

The centroid sizes were all normally distributed according to a Shapiro–Wilk test and homoscedastic according to Levene’s test; thus, the Student’s *t*-test was used to assess the differences between the size of male and female faces. The global centroid size of males always proved larger than that of females, even though a statistically significant difference in the size of the face was only observed from late childhood to adolescence. The average centroid sizes for males and females and the related *p*-value are reported in Table 4.

The multivariate regression showed that the influence of facial size on the shape was significant from late childhood (7 to 12 years old) to following age classes, as summarized in Table 5.

**Figure 3:**
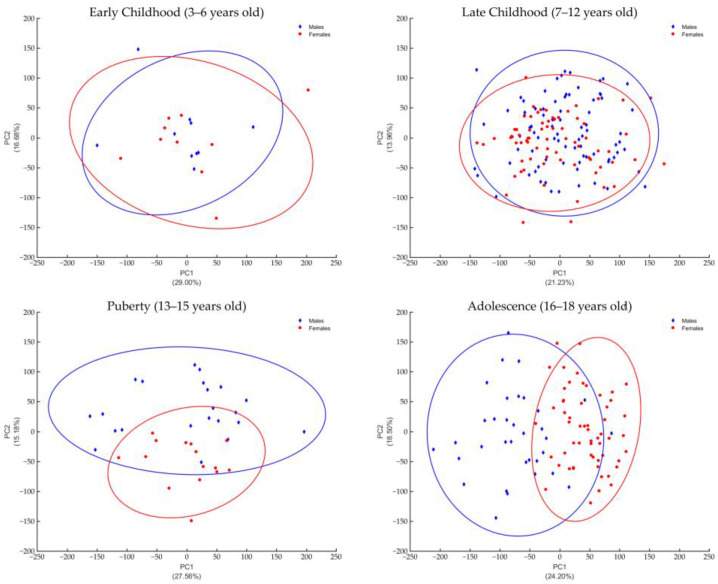
Plots of the first and second principal components with a 95% confidence interval.

The facial shape variation in the age classes was evaluated by Principal Component Analysis (PCA) coupled with parallel analysis to determine the number of significant PCs. The number and related percentage of variance explained by each of the retained PC for each age class is reported in Table 6. The effect of the first and second principal components on the facial shape are depicted as scatter plots in Figure 3.

The first and second principal components (PCs), when considered together, were responsible for more than 40% of the total variability in facial shape in all age classes, except for the late childhood one (35% of total shape variability). The PCA scatter plots (Figure 2) depict the relationship between PC1 and PC2, showing the distribution of males and females in each age class. The variation in facial shape was greater for boys in all age classes, as testified by the greater dispersion of the points and larger 95% confidence ellipses, except for the late childhood age class, where males and females had a similar variability. Additionally, the PCA scatter plots demonstrate a high degree of overlap between males and females in the first two age classes, meaning that boys and girls can not be distinguished based on what concerns the PCs under consideration. In puberty, PC2 slightly separates males and females, which becomes more evident along the PC1 in the adolescent age class. The Procrustes distance between the average male and female facial shape was significant in all age classes, as reported in Table 7, meaning that the two shapes are different on average.

**Figure 4:**
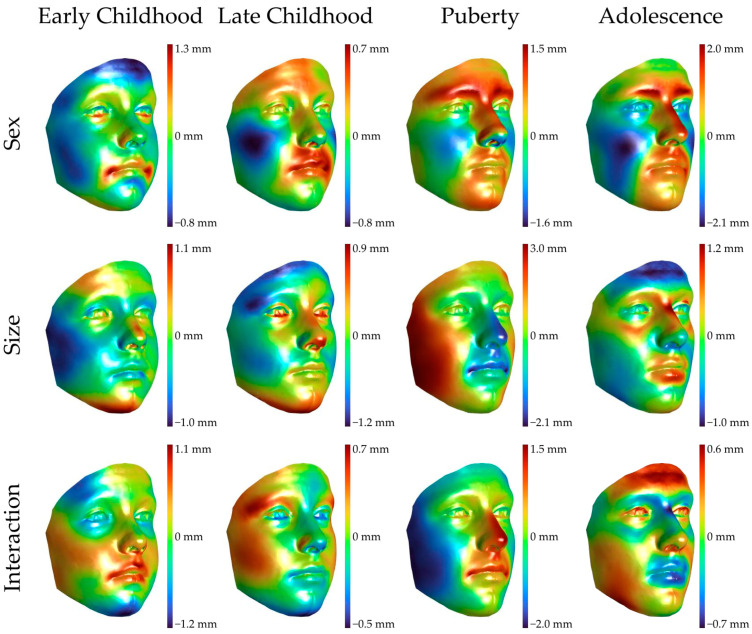
Effects of sex, size, and the interaction on the facial shape. Sex evaluates the female-to-male transition, while size is from narrower to larger faces (centroid size).

The results of the Partial Least Square Regression (PLSR) of the predictor variables (i.e., sex, size, and their interaction) for each age class are reported in Table 8, while Figure 4 shows the effect of each predictor on the facial shape morphology of each age class.

In early childhood (3 to 6 years old), none of the predictors (sex, size, and interaction) had a significant effect on the shape morphology, although some peculiar features can be observed as males are characterized by a pronounced retrusion of the forehead, flatter cheeks, deeper mento-labial sulcus, and a protrusion of the lips. In late childhood, all three predictors have a significant effect on the morphology of the facial soft tissues but with a very small impact, as demonstrated by the partial R^2^ value. In this age class, sex accentuates the flattening of the cheeks in the male sample and the protrusion of the lips that extends to the perioral region, particularly in the area included between the nasolabial sulci and the labial philtrum. The feature of the forehead retrusion is lost and reverses, with a mild protrusion in the male population. The magnitude of differences in this age class ranges from −0.8 to 0.8 mm, with the greatest effects affecting the perioral region and the cheeks.

In puberty, all the predictors have a significant effect on the facial shape. However, in this specific case, the contribution of sex, size, and their interaction is much greater than in the previous age classes, as they, respectively, contribute 9.83%, 12.20%, and 10.90% to the shape variability. The sexually dimorphic features visible during late childhood are very exaggerated, as explained by the huge increase in the partial R^2^ values in comparison to the previous ages, as well as by the increased amplitude of the color bar that ranges from −1.6 to 1.6 mm. The protrusion of the superior part of the forehead is still present, but the greatest changes occur at the level of the glabella and brow ridges, where males protrude up to 1.6 mm compared to females. The flatter profile of the cheeks and the protrusion of the lips and perioral region, pertaining to the chin region and extending to the medial lower third of the face, are also confirmed in pubertal males. In puberty, nasal sexual differences become evident, with males having a more protruded nasal dorsum and larger nose in general but with a flatter nasal base and columella. Lastly, the facial morphology of adolescents is highly influenced by sex (R^2^ = 11.82%), whilst the size and the interaction between sex and size lost significance. In adolescent faces, the sexually dimorphic traits that originated during childhood further develop in this age class, reaching the highest expression prior to adulthood. The prominence of the glabellar and brow ridges regions of males reaches a 2 mm protrusion compared to the female ones, as well as the nasal dorsum. Milder effects (around 1 mm) are observed in the protrusion of the oral and peroral soft tissues in males. Males are also characterized by a flatter profile of the cheeks that also extends downward in the lower third of the face and deeper-set eyes compared to females.

The contribution of sex, found to be statistically significant from the 7th year of life, is evidenced by the increasing values of R^2^ (variance attributable to sex) in the facial shape morphology and visually recognizable by the expansion and increasing magnitude of blue and red areas in the color-coded maps (Figure 3) representing the transition from the average female to the average male 3D models.

## 4. Discussion

The study of facial sexual dimorphism in juveniles provides useful insights into how the development of morphological differences between the two sexes develops across childhood and adolescence. These insights are particularly relevant in fields such as social and psychological research, genetics, evolutionary biology, and developmental studies, as well as in applied contexts like forensics, anthropology, and clinical practice. A deeper understanding of facial morphology and its characteristics, such as sexual dimorphism, has been possible thanks to two paired factors [10,23]: (i) the greater availability of three-dimensional imaging technologies/instruments, which are commonplace in research and clinical settings today and have allowed for the creation a large dataset of 3D models [43,45], and (ii) the introduction of advanced analytical methods, such as geometric morphometrics. This modern approach to facial analysis has allowed a deeper understanding and characterization of sexual dimorphism in diverse ages ranging from infancy to adulthood [4,5,9,10,21,24,39,88,89,90].

In our study, we employed a spatially dense geometric morphometric analysis to identify the patterns of facial sexual dimorphism in developing subjects and to examine its potential changes within an Italian population from early childhood (ages 3–6 years) through late childhood (ages 7–12 years) to puberty (ages 13–15 years) until adolescence (ages 16–18 years). The qualitative and quantitative results contributed to the establishment of age- and sex-specific craniofacial three-dimensional norms, which have important applications in forensics and clinical settings [21].

Overall, our results demonstrated the existence of numerous sex-related differences in the facial shape and size of subadults, which are already observable in early and late childhood (3 to 6 and 7 to 12 years old) and, thus, prior to puberty as already reported in the literature [4,22,23,24]. The pattern of size dimorphism between the two sexes emerged at 7 years old when centroid sizes proved significantly different. Male subjects consistently demonstrated larger average centroid sizes even in the following age classes, suggesting that postnatal growth trajectories differ between sexes: females experience growth spurts and reach their adult size prior to males, who, in turn, continue their development up to early adulthood [91,92,93,94,95]. The differences in the growth trajectories contribute to explaining the morphological variability observed in the facial shape described afterward.

Sexual dimorphism, despite the importance of the size, was also characterized by differences in the overall facial shape and its structures. Procrustes distance revealed significant shape differences in all age classes, while the Partial Least Square Regression proved notable sexual dimorphism from late childhood. This discrepancy is likely due to the different analytical goals of each method: Procrustes distance captures average shape differences, whereas PLSR identifies the predictive relationship between shape and sex. In early childhood (3 to 6 years old), only the Procrustes distance found significant differences between the average male and female facial shapes, while the PLSR did not. Thus, other factors such as genetics or age may be stronger contributors to facial morphology in this age class. Biological sex becomes a more significant predictor of aging, and our results support this hypothesis as sex becomes a significant predictor in the following age classes, and its contribution to facial morphology (R^2^) increases with age.

Our results concerning the Procrustes distance are in accordance with those reported by Kesterke et al. [4], who, using the same statistics, found significant differences between the average male and female 3D facial shapes in the same age classes defined in our study [4]. In addition, as in Kesterke et al. [4], we found the Procrustes distance to increase when moving toward older age classes except for late childhood, where a smaller value of distance is observed. Smith et al. [89] used the Procrustes distance to evaluate the sexual dimorphism of the entire head when comparing broader age groups (3 to 10 and 11 to 20 years old) for both shape and form. The authors observed an increase in the Procrustes distance moving from the younger to the older group but revealed significant distances— and so sexual dimorphism—only for the shape and form in prepuberty and adolescence (11–20 years old). The differences between our and their results, which is the lack of shape and form of sexual dimorphism in the younger age group, may be related to the different age classes (Smith et al. [89] analyzed broader age categories, including early and middle childhood together), the different area analyzed (we analyzed only the face instead of the entire head), and the small sample size in the younger age category as stated by the authors themselves. The evaluation of the color-coded maps (Figure 3) obtained through the PLSR allows us to localize and quantify shape differences due to a variable of interest, namely the biological sex. Male children of 3 to 6 years old children showed more retrusive foreheads, deeper mento-labial sulcus, protrusive lips, and less rounded faces due to the less protrusive cheeks and temporal regions. Matthews et al. [23] made similar observations in a sample size of subjects aged 0.05 to 18.60 years using an approach analogous to ours. The authors [23] reported the retro-positioning of the forehead in males to be already present as early as 1.12 years old and upper lip sex differences to emerge at 3 years old. While Matthews et al. [23] found that chin differences are noticeable at 8 years old, we find a different depth of the mento-labial sulcus in early childhood, in accordance with Kesterke et al. [4]. Kočandrlovà et al. [22] longitudinally evaluated children from 3 to 6 years old, analyzing both the shape and the form (i.e., size and shape) with a spatially dense geometric morphometric study. They observed significant differences in the form but not the shape through “significance” facial maps, which are color-coded maps highlighting significant differences in the vertex positions between the average male and female 3D models: male children always had larger areas in the lateral part of the forehead, supraciliary arches, nose, lips, and perioral regions and parts of the lower face. Contrarily, Zhong et al. [79] found no facial sexual dimorphism prior to the age of 6 years. Similarly, in our study, although we found significant shape differences in early childhood through Procrustes distance, a pronounced sexual dimorphism appeared in late childhood (from 7 years) to become more distinct and evident during puberty and adolescence. In late childhood, the biological sex only explained 1% of the total facial morphology variance, and male-specific traits consolidated or emerged from the previous age class: the medial part of the face, including the nose and perioral region, and the forehead protruded, while flattening of the cheeks was observed. Kau et al. [66], in subjects aged 11 to 14 years old, partially overlapping with our late childhood and puberty age classes- identified the glabellar and nasal prominence in males and the prominence of the cheeks in females as we did, but they also noted females to have more prominent eyes and cheeks that are non-evident or in contrast with our findings. These inconsistencies could be due to methodological differences, as Kau et al. superimposed average 3D models rather than analyzing dense geometric data. Pubertal subjects are the ones where the sexually dimorphic features manifest with a greater entity, and the biological sex explains a great percentage of facial morphology variability (9.8%). The pronounced effect of sex on facial morphology reflects the great influence of pubertal sex hormones on craniofacial development: testosterone and estrogen contribute to masculinization and feminization of the face, respectively. The dimorphic traits that emerge in late childhood evolve and strengthen in this growth phase, with protrusive and retrusive features becoming even more outwardly and inwardly displaced, respectively. Our dimorphic characteristics align with those of Koudelová et al. [10] and Toma et al. [11], as they both reported males to have deeper-set eyes, flatter cheeks, and more protruded noses and chins. Koudelová et al. [10] also reported more prominent eyebrow ridges with latero-medial development in males. A possible explanation for these accentuated sex differences, besides the contribution of sex hormones that drive morphological changes in “opposite” direction inducing masculinization and feminization, is that females, at this stage, have almost or entirely completed their craniofacial development reaching their adult size morphology. Contrarily, the development of males is continuous and will complete several years later in early adulthood. This hypothesis may also explain why the contribution of sex further increases during adolescence in our sample. In this age class, the biological sex accounts for nearly 12% of facial shape variance, similar to that reported by Claes et al. [78] for adults when facial shape was considered (12.9%). A possible speculation, as we did not evaluate an adult population, is that by the age of 16–18 years old, the sexually dimorphic traits have almost reached their full expression. In our study, the dimorphic features emerged during (late) childhood and evolved through puberty and adolescence to be comparable and in accordance with the ones reported in the literature for the adult population [4,9,10,11,12,13]. Considering our results, this study adds to the growing body literature, demonstrating that sexual dimorphism is present prior to puberty and that these sex differences continue to develop through growth. These findings suggest that adult sexual dimorphism is not completely attributable to pubertal sex hormones, but it could also be due to uterine hormone levels, particularly testosterone [96,97], or sex-linked characteristics [23] such as odontometric parameters that may hugely impact the lower part of the face [98]. However, in accordance with the literature, greater changes occur with the onset of puberty.

The thorough characterization of sex-specific facial features and their evolution/changes in subjects aged 3 to 18 years old holds significant importance in forensic and clinical settings. In the former field, sex differences are crucial for both sex and age estimation [99] since morphological differences are observed at different times. These findings may find a practical application when referred to for juveniles in cases of suspected child pornography [100,101,102], asylum seekers, or for facial reconstruction/approximation in cases of long-term missing individuals to model the changes of the craniofacial soft tissues according to the estimated age [19,21,103]. In clinical practice, sexual dimorphism and its development find way more applications, particularly in those disciplines dealing with the craniofacial districts (such as cranio-maxillofacial surgery, dentistry, genetics, and clinical dysmorphology) where reference norms are required and they must be sex-, age-, and ethnicity-specific [104]. In genetic and clinical dysmorphologies, the knowledge of facial sex differences is crucial for the accurate diagnosis and management of syndromes and developmental disorders. For example, there are more than 10,000 genetic and rare diseases affecting 7% of the world’s population [105,106]. A peculiar craniofacial phenotype is observed in 40% of these diseases [107,108], and they range from subtle facial anomalies to severe malformations [105]. These manifestations are usually observed in infancy and childhood, and the morphological comparison with normative reference data would be beneficial for a precocious diagnosis, to estimate the entity of the dysmorphology, and to eventually guide treatments and surgical interventions (if available).

In the era of personalized medicine, it is important to know sex differences because this allows clinicians to plan interventions, either surgical or orthopedic, tailored to the specific characteristics of the patients in terms of age and sex [89,109], ensuring treatments that align with the natural developmental trajectory expected within each sex, but also to ensure long-term results and reduce the possibility of future adjustments. In addition, the knowledge of how male and female structures develop allows us to design procedures that restore the natural appearance, leading to outcomes that are both age- and gender-appropriate [4,45], as in the case of several craniofacial anomalies and syndromes.

### Advantages and Limitations

Our study, besides taking advantage of a spatially dense morphometric approach allowing the analysis of the entire facial surface, contrarily to the traditional anthropometric ones, also benefited from the creation of a personalized facial “template” to overcome one of the main limitations of the template provided in the MeshMonk toolbox: the lack of the most postero-lateral part of the face including the gonial region or upper part of the forehead up to the hairline. Consideration is worthwhile due to the potential impact of population-specific traits on the manifestation of sexual dimorphism. Our study, to the best of our knowledge, is the first one to provide valuable insights about facial sexual dimorphism in an Italian subadult population through a spatially dense geometric morphometric approach. However, it is known that sexually dimorphic traits can vary among different populations [5], as is the case with the ones referenced and discussed in this study. Thus, future studies should aim to systematically analyze and evaluate these variations.

A first limitation is the cross-sectional nature of this study, potentially affecting the results since different subjects have been analyzed for each age class and do not actually represent real growth, even though the use of average models allows the generalizability of the results. Another limitation is the limited sample size of the first age class, which might have misled the results, preventing their robust interpretation and generalizability. Due to the controversies about the results in children this young, it would be auspicious to implement the sample size in further studies to more accurately and robustly assess the possible existing differences in early childhood.

## 5. Conclusions

This study contributes to a growing body of evidence that sex-related differences in facial morphology emerge in early childhood and become more pronounced with age, especially during puberty, before later stabilizing in adolescence. Our spatially dense approach offered a refined understanding of these dimorphic traits, demonstrating differences even in pre-pubertal age groups. Future research should aim to standardize methodologies across studies and consider broader, geographically diverse samples to further elucidate patterns of facial sexual dimorphism in subadults. This work not only challenges traditional views but also emphasizes the utility of advanced morphometric approaches in uncovering subtle developmental patterns in craniofacial morphology, particularly referencing sexual dimorphism. The presented results are also useful in clinical and forensics contexts as sex- and age-specific reference data in diverse clinical and forensic settings.

## Figures and Tables

**Table 1:** Name and definition of anthropometric landmarks used in the first step of the non-rigid alignment.

Unpaired Median Landmarks		
Name	Abbreviation	Definition
Trichion	tr	The point on the hairline in the midline of the forehead
Nasion	n	The point in the midline of both the nasal root and the nasofrontal suture
Pronasale	prn	The most protruded point of the apex nasi
Subnasale	sn	The midpoint of the angle at the columella base where the lower border of the nasal septum and the surface of the upper lip meet
Sublabiale	sl	The point in the midline of the mentolabial ridge
Gnathion	gn	The lowest median landmarks on the lower border of the mandible
Paired bilateral landmarks		
Name	Abbreviation	Definition
Frontotemporale	ft	The point on each side of the forehead, laterally from the elevation of the linea temporalis
Zygion	zy	The most lateral point of each zygomatic arch
Tragion	t	The notch on the upper margin of the tragus
Gonion	go	The most lateral point on the mandibular angle

**Table 2:** Sample distribution according to age and sex.

Age Group		Male	Female	Total	*p*-Value
Early Childhood (3–6 y.o.)	*n*	10	10	20	0.741
	Mean age	5.78	5.92	5.85	
Late Childhood (7–12 y.o.)	*n*	83	73	156	0.068
	Mean age	10.82	10.28	10.55	
Puberty (13–15 y.o.)	*n*	22	15	37	0.135
	Mean age	14.01	14.46	14.24	
Adolescence (16–18 y.o.)	*n*	34	57	91	0.768
	Mean age	17.88	17.89	17.89	
Total (3–18 y.o.)	*n* Mean age	149 12.12	155 12.14	304 12.13	

y.o.: years old.

**Table 3:** Intraclass correlation coefficient (ICC) of the intra- and inter-operator reliability of landmarks indication for the three axes.

	Intra-Operator Reliability			Inter-Operator Reliability		
Landmark	X	Y	Z	X	Y	Z
tr	0.975	0.980	0.998	0.955	0.968	0.997
*n*	0.985	0.990	0.996	0.975	0.989	0.996
prn	0.995	0.998	0.939	0.986	0.997	0.966
sn	0.991	0.995	0.938	0.985	0.991	0.961
sl	0.989	0.995	0.993	0.969	0.994	0.995
gn	0.950	0.996	0.993	0.940	0.985	0.989
t_(r)_	0.991	0.994	0.996	0.989	0.997	0.965
t_(l)_	0.992	0.994	0.959	0.977	0.986	0.954
ft_(r)_	0.972	0.970	0.974	0.991	0.994	0.987
ft_(l)_	0.943	0.938	0.966	0.963	0.967	0.977
zy_(r)_	0.969	0.951	0.991	0.950	0.938	0.991
zy_(l)_	0.980	0.917	0.981	0.960	0.927	0.978
go_(r)_	0.991	0.988	0.977	0.988	0.986	0.992
go_(l)_	0.942	0.952	0.983	0.965	0.967	0.997

(r): right; (l): left.

**Table 4:** Average centroid size for males and females.

Age Class	Male Average (SD)	Female Average (SD)	*p*-Value
Early Childhood	4125.12 (166.20)	4077.61 (189.63)	0.559
Late Childhood	4483.37 (198.75)	4344.03 (185.89)	<0.001 *
Puberty	4798.73 (183.32)	4667.29 (219.34)	0.028 *
Adolescence	4928.30 (148.68)	4674.60 (162.01)	<0.001 *

*: significant for α = 0.05.

**Table 5:** Static allometry.

Age Class	Wilk’s λ	*p*-Value
Early Childhood	0.67544	0.181
Late Childhood	0.63616	<0.001 *
Puberty	0.63554	<0.001 *
Adolescence	0.63938	<0.001 *

*: significant for α = 0.05.

**Table 6:** Percentage of variance explained by each principal component.

	Early Childhood		Late Childhood		Puberty		Adolescence	
PC	Variance (%)	Eigenvalues	Variance (%)	Eigenvalues	Variance (%)	Eigenvalues	Variance (%)	Eigenvalues
1	29.00	5550.35	21.23	4220.12	27.56	6346.45	24.20	5514.12
2	16.68	3192.57	13.96	2774.99	15.18	3494.95	18.50	4215.47
3	11.05	2115.75	9.57	1902.86	12.79	2946.58	9.83	2239.13
4	9.02	1726.42	7.49	1488.1	9.47	2181.67	7.84	1786.39
5			6.30	1251.54	6.28	1445.78	5.36	1221.34
6			5.64	1120.77	3.68	846.65	3.67	836.25
7			3.67	729.23	3.35	770.66	3.15	717.75
8			3.32	659.75			2.66	605.44
9			2.43	482.26			2.22	506.71
10			2.17	431.19			1.94	442.84
11			1.99	394.66			1.74	397.15
12			1.83	362.86			1.69	385.81
13			1.49	296.48			1.47	335.05
14			1.33	263.74				
15			1.27	251.77				
16			1.10	218.44				
17			0.92	182.89				
Total	65.75		85.61		78.31		84.27	

**Table 7:** Procrustes distance and related *p*-value tested under 10,000 permutations.

	Procrustes Distance	*p*-Value_10,000_
Early Childhood	0.00026	<0.001 *
Late Childhood	0.00011	<0.001 *
Puberty	0.00077	<0.001 *
Adolescence	0.00143	<0.001 *

*: significant for α = 0.05.

**Table 8:** Significance and contribution of sex, size, and interaction on the facial shape variance.

	Sex		Size		Sex*Size	
	*p*-Value_10,000_	Partial R^2^	*p*-Value_10,000_	Partial R^2^	*p*-Value_10,000_	Partial R^2^
Early Childhood	0.699	0.0399	0.192	0.0698	0.432	0.0524
Late Childhood	0.027 *	0.0127	<0.001 *	0.0114	0.026 *	0.0131
Puberty	<0.001 *	0.0983	<0.001 *	0.1220	0.002 *	0.1090
Adolescence	<0.001 *	0.1182	0.156	0.0153	0.292	0.0129

*: significant for α = 0.05.

## Data Availability

The data presented in this study are available upon reasonable request from the corresponding author.
